# Post-hospitalization dialysis facility processes of care and hospital readmissions among hemodialysis patients: a retrospective cohort study

**DOI:** 10.1186/s12882-018-0983-5

**Published:** 2018-07-31

**Authors:** Laura C. Plantinga, Tahsin Masud, Janice P. Lea, John M. Burkart, Christopher M. O’Donnell, Bernard G. Jaar

**Affiliations:** 1 Division of Renal Medicine, Department of Medicine, Emory University, Atlanta, GA USA; 2 Department of Epidemiology, Rollins School of Public Health, Emory University, Atlanta, Georgia USA; 3 Wake Forest Baptist Medical Center, Winston-Salem, North Carolina USA; 4 Department of Medicine, Johns Hopkins School of Medicine, Baltimore, MD USA; 5 Welch Center for Prevention, Epidemiology and Clinical Research, Johns Hopkins University, Baltimore, MD USA; 6 Department of Epidemiology, Johns Hopkins Bloomberg School of Public Health, Baltimore, MD USA; 7 Nephrology Center of Maryland, Baltimore, MD USA

**Keywords:** Readmission, Rehospitalization, Pulmonary edema, Fluid overload, Dialysis

## Abstract

**Background:**

Both dialysis facilities and hospitals are accountable for 30-day hospital readmissions among U.S. hemodialysis patients. We examined the association of post-hospitalization processes of care at hemodialysis facilities with pulmonary edema-related and other readmissions.

**Methods:**

In a retrospective cohort comprised of electronic medical record (EMR) data linked with national registry data, we identified unique patient index admissions (*n* = 1056; 2/1/10–7/31/15) that were followed by ≥3 in-center hemodialysis sessions within 10 days, among patients treated at 19 Southeastern dialysis facilities. Indicators of processes of care were defined as present vs. absent in the dialysis facility EMR. Readmissions were defined as admissions within 30 days of the index discharge; pulmonary edema-related vs. other readmissions defined by discharge codes for pulmonary edema, fluid overload, and/or congestive heart failure. Multinomial logistic regression to estimate odds ratios (ORs) for pulmonary edema-related and other vs. no readmissions.

**Results:**

Overall, 17.7% of patients were readmitted, and 8.0% had pulmonary edema-related readmissions (44.9% of all readmissions). Documentation of the index admission (OR = 2.03, 95% CI 1.07–3.85), congestive heart failure (OR = 1.87, 95% CI 1.07–3.27), and home medications stopped (OR = 1.81, 95% CI 1.08–3.05) or changed (OR = 1.69, 95% CI 1.06–2.70) in the EMR post-hospitalization were all associated with higher risk of pulmonary edema-related vs. no readmission; lower post-dialysis weight (by ≥0.5 kg) after vs. before hospitalization was associated with 40% lower risk (OR = 0.60, 95% CI 0.37–0.96).

**Conclusions:**

Our results suggest that some interventions performed at the dialysis facility in the post-hospitalization period may be associated with reduced readmission risk, while others may provide a potential existing means of identifying patients at higher risk for readmissions, to whom such interventions could be efficiently targeted.

**Electronic supplementary material:**

The online version of this article (10.1186/s12882-018-0983-5) contains supplementary material, which is available to authorized users.

## Background

Among the more than 400,000 prevalent end-stage renal disease (ESRD) patients being treated with hemodialysis in the United States, hospitalizations are frequent and account for ~ 40% of all Medicare dialysis expenditures [1]. Furthermore, more than one-third of hospitalizations among U.S. hemodialysis patients result in a readmission within 30 days of discharge [1]. We previously showed that nearly half (44%) of the readmissions among U.S. dialysis patients were related to pulmonary edema, which can be seen in the setting of congestive heart failure (CHF) and fluid overload in dialysis [2]. While several characteristics of the index hospitalizations were strongly associated with higher risk of these common pulmonary edema-related readmissions, we found that only a few patient characteristics (e.g., history of CHF and documented dialysis non-adherence) were associated with these readmissions [2].

In an effort to reduce hospital readmissions and associated costs specifically among U.S. dialysis patients, the Centers for Medicare & Medicaid Services (CMS) now holds both hospitals and dialysis facilities accountable for higher-than-expected readmission risks [3, 4]. Standardized readmissions ratios are included in the publicly reported 5-star ratings of both hospitals [5] and dialysis facilities [6], and these ratings are tied to reimbursement. Despite this, the reasons for readmissions among dialysis patients remain underexplored [7–15]. Particularly, it remains unknown whether usual processes of care at the dialysis facility [14, 16–19] in the post-hospitalization period help reduce risk of pulmonary edema-related readmissions, thus decreasing the risk of readmissions overall. Leveraging electronic medical record (EMR) data from 19 Southeastern not-for-profit dialysis clinics linked with detailed national administrative data on hospital admissions, we aimed to estimate associations of post-hospitalization dialysis facility processes of care with pulmonary edema-related and other 30-day readmissions among hemodialysis patients.

## Methods

### Study population and data sources

Data for this study were obtained from the EMRs of the dialysis clinics operated by Emory Healthcare (*n* = 3 clinics) and Wake Forest Baptist Health (*n* = 16 clinics) and from the United States Renal Data System (USRDS) [1], with approval and oversight for data from both sites provided by the Emory Institutional Review Board. Patients in the EMR data were linked to USRDS via identifiers including names, Social Security numbers, and dates of birth. In the prevalent cohort of Emory and Wake Forest patients, index hospitalizations (first hospitalization after ≥90 days on in-center hemodialysis at Emory or Wake Forest) were identified in the period from 2/1/10 (when Emory clinics opened) to 7/31/15, using the linked USRDS hospitalization file. We identified 1945 index hospitalizations in this follow-up period. Index admissions were excluded if the patient was not documented in the USRDS as being on hemodialysis at admission (*n* = 168) or for at least 30 days after discharge (*n* = 89), was < 18 or > 100 years old (*n* = 1), and or did not have primary Medicare coverage in the 30 days after index discharge (to ensure complete capture of hospital admissions; *n* = 410). For examination of post-discharge dialysis facility processes of care, we also excluded *n* = 221 index admissions that were not followed by at least three outpatient dialysis sessions documented in the Emory/Wake Forest EMR in the 10 days after discharge. This exclusion allowed for the examination of effects of dialysis facility processes of care only in cases for which dialysis providers had the time and opportunity to clinically intervene as necessary. The final study population included 1056 index admissions.

### Study variables

#### Readmissions

Readmissions were defined using the linked USRDS hospital file as hospital admissions that occurred within 30 days of the index admission discharge. In primary analyses, pulmonary edema-related readmissions were identified via discharge International Classification of Diseases, Ninth Revision (ICD-9) codes of fluid overload (276.6, 276.61, or 276.69), heart failure (428.x, 402.x1, 404.x1, 404.x3, or 398.91), or pulmonary edema (518.4 or 514), in any position [20, 21].

#### Dialysis facility processes of care

Post-discharge processes of care were captured in the Emory/Wake Forest EMR data in the three dialysis sessions immediately following discharge. Index admission documentation was defined as a record of the index admission (EMR date of admission within 3 days of admission date noted by USRDS) in the Emory/Wake Forest EMR. Documentation of attributed cause of hospitalization was defined as a cause other than “none” noted in the EMR record of the hospitalization. Documentation of CHF was defined as the presence of CHF in the EMR problem list at the time of index discharge (among *n* = 621 patients with CHF as defined in the USRDS). Drawing of labs was defined as the record of any lab test ordered (as documented in the EMR) within three sessions after index discharge; draws for albumin and hemoglobin/hematocrit within this time frame were also considered separately. Target weight decrease was defined as a decrease in weight of ≥0.5 kg documented in dialysis orders within the first three post-index discharge dialysis sessions, relative to the documented target weight in the dialysis facility EMR prior to index hospitalization; actual weight decreases of ≥0.5 kg were also examined. Higher erythropoietin-stimulating agent dose was defined as an EMR-documented dose in the first three post-index dialysis sessions that was higher than the previous dose ordered prior to hospitalization. A discontinuation of home medications was defined as any home medication with a stop date on the dialysis facility EMR home medication list within the first three dialysis sessions of discharge from the index hospitalization. Changes in home medications included both discontinued and added medications within three sessions. Current ultrafiltration rate policy in place at the treating facility at the patient’s index discharge was also examined in sensitivity analyses. This policy, enacted in 4/2012 at 15 of the facilities, required providers to lengthen prescribed treatment time in increments of 15 min (up to 1 h) for any session in which the patient’s anticipated ultrafiltration rate (given intradialytic weight gain) was > 13 ml/kg/hour.

#### Covariates

Patient age and ESRD vintage at index admission were calculated using the differences between date of admission and dates of birth and first ESRD service available in the linked USRDS data. Race/ethnicity and assigned cause of ESRD were obtained from the Medicare ESRD eligibility form (CMS-2728) data available in USRDS. Comorbid conditions were considered pre-existing if they appeared on the CMS-2728 or were present in discharge codes from all hospital discharges in the year up to and including the index admission, using the diagnostic codes outlined in the CMS Chronic Conditions Warehouse algorithms [22]. History of dialysis non-adherence was assessed during the same time period as the comorbid conditions, using ICD-9 code V45.12 (noncompliance with renal dialysis). For the index admission, length of stay was calculated as the discharge date minus admission date. Intensive care utilization was determined by whether patients spent ≥1 day in an intensive care or coronary care unit during the index admission.

### Statistical analysis

Patient characteristics were summarized as means and standard deviations (SDs), medians and interquartile ranges (IQRs), or percentages, as appropriate. The burden of readmissions was determined as the percentage of index admissions that resulted in a readmission within 30 days of discharge from the index admission, either overall or attributed to pulmonary edema, using the primary definition described above. Patients with and without three dialysis sessions in the 10 days following index discharge were compared. Crude risks of readmission (both pulmonary edema-related and other) were compared by dialysis facility processes of care using chi-square tests. Multinomial logistic regression models were used to estimate adjusted odds ratios (ORs) for both pulmonary edema-related and other readmissions, vs. no readmissions, by dialysis facility processes of care. We additionally adjusted for characteristics significantly associated with readmissions, selected from an a priori-identified list of patient and index admission characteristics that might affect risk of readmission, as informed by our previous work [2]. Complete case analysis was used for all models. In secondary analyses, we stratified results by CHF and diabetes and also examined two dichotomized outcomes (any vs. no readmission and pulmonary edema-related readmission vs. no pulmonary edema-related readmission) using multivariable logistic regression models to estimate ORs. All analyses were performed with Stata v 14.2 (College Station, TX). The statistical significance threshold was set at α = 0.05.

## Results

### Characteristics of patients, index admissions, and readmissions

**Fig. 1:**
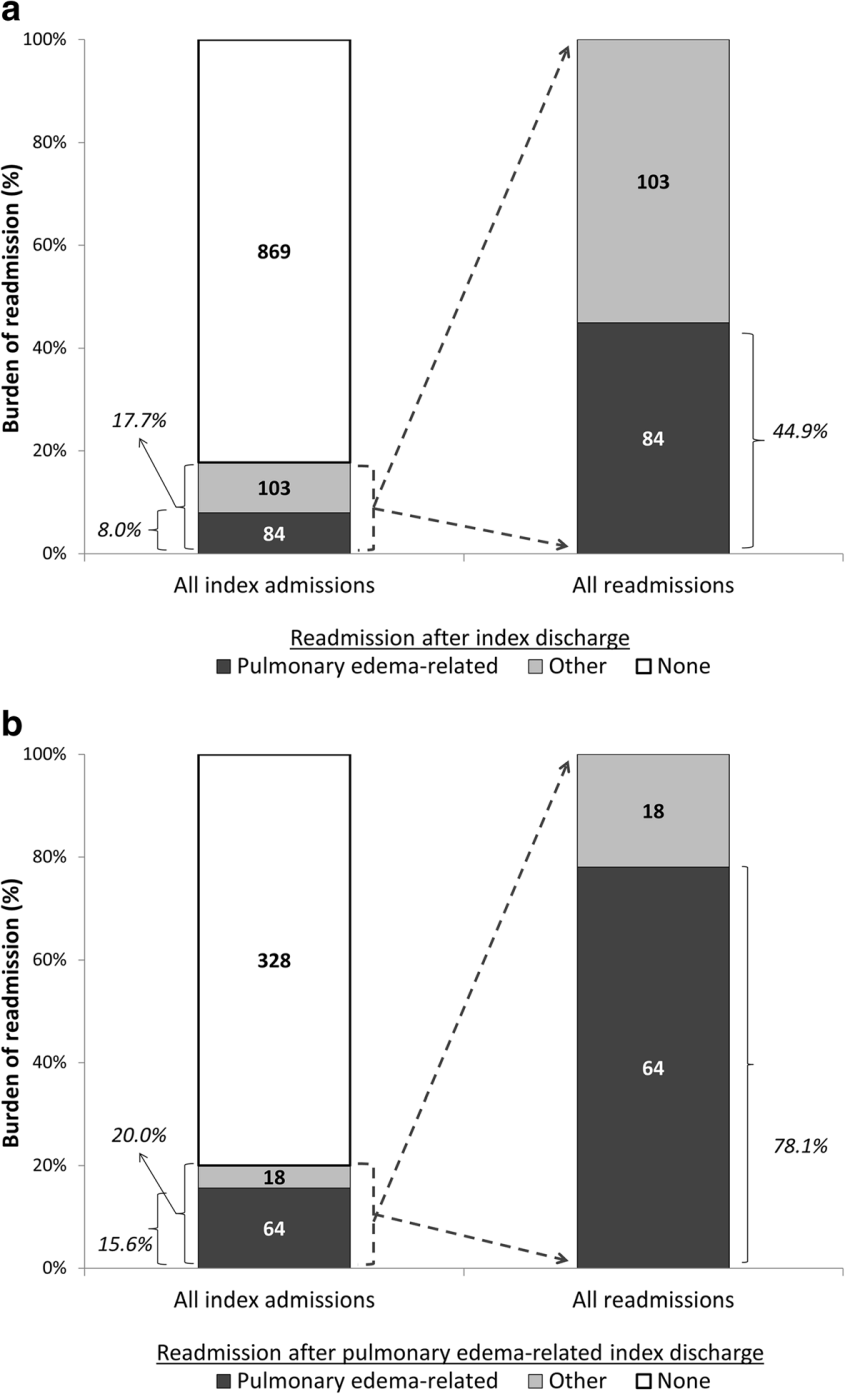
Overall and pulmonary edema-related readmissions within 30 days of any index admission (*N* = 1056) (**a**) and within 30 days of pulmonary edema-related index admission (*N* = 410) (**b**), among hospitalized Emory and Wake Forest hemodialysis patients in 1/2010–7/2015

Among the 1056 index admissions included in the study, 189 (17.7%) were followed by a 30-day readmission; of these, 44.9% were related to pulmonary edema (Fig. 1a). Among 410 index admissions related to pulmonary edema, 20.0% were followed by a readmission, 78.1% of which were also pulmonary edema-related (Fig. 1b). Overall, index admissions had a median length of stay of 4 days, 38.8% were pulmonary edema-related, and 23.9% involved intensive care utilization. While index admissions followed by pulmonary edema-related readmissions were far more likely to be related to pulmonary edema than those followed by other readmissions or no readmissions (76.2% vs. 17.5 and 37.7%, respectively), there were no differences in length of stay or intensive care use by readmission status (Table 1). Patients who had pulmonary edema-related readmissions were slightly older (62.4 vs. 58.5 and 60.4) and more likely to white (45.2% vs. 32.4 and 33.0%) than those with other or no readmissions, but these differences were not statistically significant. Those with pulmonary edema-related vs. other and no readmissions were statistically significantly more likely to have documented history of non-adherence (7.1% vs. 5.8 and 2.6%) and history of CHF (73.8% vs. 28.2 and 40.8%). Results with dichotomous exposures (any vs. no 30-day readmission and pulmonary edema-related vs. no pulmonary edema-related readmission) were similar (Additional file 1: Table S1).

**Table 1:** Index admission and patient characteristics of a cohort of prevalent hemodialysis patients admitted at least once while on hemodialysis treatment at Emory or Wake Forest between January 2010 and July 2015, by no, pulmonary edema-related, and other 30-day readmission status

Characteristic	Overall	30-day readmission status			P*
		None	Pulmonary edema-related readmission	Any other readmission	
*N*	*1056*	*869 (82.3%)*	*84 (8.0%)*	*103 (9.8%)*	*–*
Index admission** characteristic					
Median length of stay (IQR), days	4 (2–7)	4 (2–7)	4 (2–7)	5 (2–8)	0.48
Pulmonary edema-related (%)	38.8%	37.7%	76.2	17.5%	< 0.001
Intensive care utilization (%)	23.9%	24.3%	20.2%	23.3%	0.74
Patient demographics					
Mean (SD) age, years	60.5 (15.0)	60.4 (14.9)	62.4 (15.4)	58.5 (15.3)	0.20
Female (%)	47.8%	47.2%	50.0%	51.5%	0.65
Race/ethnicity (%)					0.14
Non-Hispanic white	33.9%	33.0%	45.2%	32.4%	
Non-Hispanic black	63.5%	64.2%	54.8%	64.7%	
Hispanic or other	2.6%	2.8%	0.0%	2.9%	
Patient clinical factors					
Median (IQR) dialysis vintage, years	1.0 (0.3–3.9)	1.1 (0.3–3.8)	1.0 (0.3–4.0)	0.7 (0.2–4.5)	0.30
History of dialysis non-adherence	3.4%	2.6%	7.1%	5.8%	0.02
Primary assigned cause of ESRD (%)					> 0.9
Diabetes	40.9%	41.2%	38.1%	39.8%	
Hypertension	27.6%	27.3%	29.8%	28.2%	
Glomerulonephritis	11.4%	11.3%	13.1%	10.7%	
Other	20.2%	20.2%	19.1%	21.4%	
Comorbid conditions (%):					
Diabetes	59.8%	60.3%	54.8%	60.2%	0.62
Ischemic heart disease	27.3%	27.1%	34.5%	23.3%	0.22
Hypertension	98.9%	98.8%	98.8%	100%	0.71
Congestive heart failure	42.4%	40.8%	73.8%	28.2%	< 0.001

*BMI* body mass index, *COPD* chronic obstructive pulmonary disease, *ESRD* end-stage renal disease, *IQR* interquartile range. *N* = 1056 overall, except for race/ethnicity (*N* = 1050), assigned cause of ESRD (*N* = 1055)

^*^By ANOVA, equality-of-medians, or Fisher’s exact test, as appropriate

Comparing the 1056 included patients with the 221 patients excluded due to having < 3 sessions in the first 10 days after discharge from index admission, we found that excluded patients were more likely than those included to be readmitted (32.1% vs. 17.7%, *P* < 0.001) and to be readmitted related to pulmonary edema (13.6% vs. 8.0%, *P* = 0.008). History of dialysis non-adherence was similar in the excluded vs. included populations (3.8% vs. 3.4%, *P* = 0.8). Those excluded vs. included were less likely to be discharged to home (70.1% vs. 82.5%; *P* < 0.001) and more likely to be discharged to inpatient rehabilitation (6.8% vs. 0.8%; *P* < 0.001) after the index admission. However, excluded and included patients were similarly likely to be discharged to a skilled nursing facility (13.6% vs. 14.3%).

### Post-index hospitalization dialysis facility processes of care and readmissions

In multinomial models, we found that documentation of index admission in the dialysis facility EMR was associated with 3.3- and 2.5-fold risk of pulmonary edema-related and other vs. no readmissions; after adjustment, EMR documentation remained associated with 2-fold greater risk of pulmonary edema-related vs. no readmission (Table 2). Additionally, documentation of the cause of the index admission in the EMR was slightly higher in pulmonary edema-related vs. other and no readmissions (87.3% vs. 72.3 and 78.6%). Documentation of CHF in the problem list, among those identified as having CHF using the linked administrative data, was associated with 1.9-fold higher risk of pulmonary edema-related vs. no readmissions but 65% lower risk of other vs. no readmissions, after adjustment (Table 2). While drawing of labs was associated with greater risk of pulmonary edema-related and other (vs. no) readmissions, these associations were generally not robust to adjustment. However, post-discharge albumin draws remained associated with 1.6-fold higher likelihood of other vs. no readmission, and post-discharge hemoglobin/hematocrit draws remained associated with 1.7-fold higher risk of pulmonary edema-related vs. no readmission (not statistically significant; Table 2). Among those with albumin drawn immediately post-hospitalization, those who were readmitted due to pulmonary edema-related or other reasons vs. not readmitted had lower average albumin values (3.57 g/dl in both readmitted groups vs. 3.67 g/dl in the non-readmitted group). Post-discharge decreases in target post-dialysis weight (as noted in the dialysis orders) of ≥0.5 kg were noted for 32.1% of pulmonary edema-related index admissions, vs. 23.3 and 20.7% of index admissions followed by other and no readmissions, respectively. Decreases in actual post-dialysis weight of ≥0.5 kg after index admissions were associated with 40% lower risk of pulmonary edema-related vs. no readmissions, but were not associated with risk of any other vs. no readmission. The interactions between actual post-dialysis weight of ≥0.5 kg and index hospitalization cause (pulmonary edema vs. other) were not statistically significant for our primary outcome, but were statistically significant [*P*_*interaction*_ = 0.02, OR = 0.53 (95% CI, 0.32–0.87) vs. OR = 1.17 (95% CI, 0.77–1.77) for index admissions due to pulmonary edema vs. not], in models in which any vs. no readmission was the outcome. Discontinuation of home medications post-hospitalization discharge was associated with 81% higher risk of pulmonary edema-related vs. no readmissions; changes in home medications post-discharge (including added and discontinued medications) were associated with 69% higher risk of pulmonary edema-related vs. no readmissions (Table 2). There was no association of ESA dose with either type of readmission vs. no readmission. An ultrafiltration rate policy in place at the patient’s treating facility at discharge was associated with nearly 50% lower pulmonary edema-related and other readmissions; these associations were attenuated and not statistically significant with adjustment (Table 2). All associations were similar in magnitude and direction when additional adjustment for dialysis vintage was performed (data not shown).

**Table 2:** Associations of dialysis facility processes of care with pulmonary edema-related and other 30-day readmissions, among hospitalized Emory and Wake Forest hemodialysis patients in 1/2010–7/2015

Dialysis facility process of care^a^	No readmission	Pulmonary edema-related readmission	Other readmission	*P*
Index admission documented				
% documented	62.5%	84.5%	80.6%	*< 0.001*
Unadjusted OR (95% CI)	---^c^	3.28 (1.79–6.02)	2.49 (1.50–4.14)	*< 0.001/< 0.001*
Adjusted^b^ OR (95% CI)	---^c^	2.03 (1.07–3.85)	1.48 (0.87–2.49)	*0.03/0.1*
% patients with CHF documented in problem list at index discharge (among *n* = 389 patients with CHF history)				
% yes	39.9%	56.5%	17.2%	*0.001*
Unadjusted OR (95% CI)	---^c^	1.95 (1.12–3.39)	0.31 (0.12–0.84)	*0.02/0.02*
Adjusted^b^ OR (95% CI)	---^c^	1.87 (1.07–3.27)	0.35 (0.13–0.96)	*0.03/0.04*
% any labs drawn within 3 sessions after index discharge				
% yes	65.7%	83.3%	81.6%	*< 0.001*
Unadjusted OR (95% CI)	---^c^	2.61 (1.45–4.71)	2.31 (1.38–3.87)	*0.001/0.002*
Adjusted^b^ OR (95% CI)	---^c^	1.38 (0.74–2.56)	1.32 (0.77–2.26)	*0.3/0.3*
% albumin checked within 3 sessions after index discharge				
% yes	33.0%	36.9%	49.5%	*0.004*
Unadjusted OR (95% CI)	---^c^	1.19 (0.74–1.89)	1.99 (1.32–3.00)	*0.5/0.001*
Adjusted^b^ OR (95% CI)	---^c^	0.88 (0.54–1.42)	1.61 (1.06–2.45)	*0.6/0.03*
% hemoglobin/hematocrit checked within 3 sessions after index discharge				
% yes	59.4%	79.8%	76.7%	*< 0.001*
Unadjusted OR (95% CI)	---^c^	2.70 (1.56–4.67)	2.25 (1.40–3.63)	*< 0.001/0.001*
Adjusted^b^ OR (95% CI)	---^c^	1.65 (0.93–2.93)	1.44 (0.88–2.35)	*0.08/0.1*
% decrease in post-dialysis weight in the first 3 sessions of at least 0.5 kg				
% yes	56.6%	50.0%	55.3%	*0.5*
Unadjusted OR (95% CI)	---^c^	0.77 (0.49–1.20)	0.95 (0.63–1.43)	*0.2/0.8*
Adjusted^b^ OR (95% CI)	---^c^	0.60 (0.37–0.96)	1.10 (0.72–1.67)	*0.03/0.7*
% higher ESA dose ordered in first 3 sessions (among *n* = 282 with ESA administered)				
% yes	16.7%	15.0%	10.0%	*0.6*
Unadjusted OR (95% CI)	---^c^	0.88 (0.25–3.16)	0.56 (0.19–1.66)	*0.8/0.3*
Adjusted^b^ OR (95% CI)	---^c^	0.88 (0.24–3.27)	0.57 (0.19–1.71)	*0.8/0.3*
% any home medication discontinued in first 3 sessions				
% yes	16.1%	31.0%	20.4%	*0.002*
Unadjusted OR (95% CI)	---^c^	2.33 (1.42–3.84)	1.33 (0.80–2.23)	*0.001/0.3*
Adjusted^b^ OR (95% CI)	---^c^	1.81 (1.08–3.05)	1.20 (0.71–2.02)	*0.02/0.5*
% any home medication changed in first 3 sessions				
% yes	27.6%	41.2%	21.3%	*0.004*
Unadjusted OR (95% CI)	---^c^	1.84 (1.19–2.82)	0.71 (0.45–1.11)	*0.006/0.1*
Adjusted^b^ OR (95% CI)	---^c^	1.69 (1.06–2.70)	0.75 (0.47–1.20)	*0.003/0.2*
% index discharge occurring at facility with current ultrafiltration rate policy^d^				
% yes	30.3%	19.1%	19.4%	*0.01*
Unadjusted OR (95% CI)	---^c^	0.55 (0.31–0.96)	0.56 (0.34–0.93)	*0.04/0.03*
Adjusted^b^ OR (95% CI)	---^c^	0.75 (0.42–1.36)	0.80 (0.47–1.35)	*0.3/0.4*

*CHF* congestive heart failure, *ESA* erythropoietin-stimulating agent

^a^Assessed in the first three sessions after index discharge

^b^Adjusted models include patient history of congestive heart failure, index admission related to pulmonary edema, and history of non-adherence

^c^Base outcome in multinomial logistic regression

^d^Ultrafiltration rate policy required providers to lengthen prescribed treatment time in increments of 15 min (up to 1 h) for any session in which the patient’s anticipated ultrafiltration rate (given intradialytic weight gain) was > 13 ml/kg/hour

Results were similar when dichotomous outcomes, rather than a single categorical outcome, were used in logistic models (Additional file 2: Table S2). The association of post-dialysis weight decrease was associated with lower risk of pulmonary edema-related readmission among those with heart failure (OR = 0.49, 95% CI 0.28–0.85) but not among those without heart failure (OR = 1.10, 95% CI 0.49–2.70). Similarly, the associations of changing medications, stopping medications, and ultrafiltration rate policy in effect with pulmonary edema-related vs. no readmissions were driven by the population with heart failure (OR = 1.99, 95% CI 1.10–3.60; OR = 1.93 95% CI, 1.10–3.38; and OR = 0.66, 95% CI 0.33–1.32, respectively), with null corresponding associations among those without heart failure. Otherwise, results stratified by heart failure and by diabetes showed no substantial differences in associations.

## Discussion

In this retrospective Southeastern cohort of prevalent hemodialysis patients with index admissions in 2010–2015, we found that 17.7% of patients were readmitted; among these readmissions, 44.9% were related to pulmonary edema. Among patients with pulmonary edema-related index admissions, 20.0% were readmitted; 78.1% of these readmissions were also related to pulmonary edema. Documentation in the dialysis EMR of the index admission, documentation of congestive heart failure, and discontinuation of home medications over three dialysis sessions within 10 days discharge from an index admission were associated with 1.8- to 2.1-fold higher risk of pulmonary edema-related vs. no readmission. These associations were similar in magnitude to that of patient history of CHF with pulmonary edema-related readmission risk in the national population [2]. Lower post-dialysis weight in the three post-discharge dialysis sessions, on the other hand, was associated with 40% lower risk of pulmonary edema-related vs. no readmission. An ultrafiltration rate policy in place, which required to providers to increase prescribed dialysis time if the intradialytic weight gain would result in a rate of > 13 ml/kg/hour, was associated with 25 and 20% lower risk of pulmonary edema-related and other readmission, respectively, vs. no readmission, but these associations were not statistically significant. Drawing of serum albumin in the post-hospitalization period was associated with 1.6-fold higher risk, whereas documentation of congestive heart failure in the EMR was associated with 65% lower risk, of other vs. no readmissions.

It might be expected that greater clinical attention paid to dialysis patients in the immediate period after hospitalization would be associated with lower readmission risk. However, we showed that documentation of index admissions, documentation of congestive heart failure, and changes in home medications in this period were actually associated with higher risk of pulmonary edema-related readmissions. Similarly, drawing of albumin in this post-hospitalization period was associated with higher risk of other readmissions. These results may reflect provider recognition of patients who are at elevated risk for hospitalization and subsequent readmission due to greater disease severity. Particularly, documentation of the hospitalization in the dialysis facility EMR may reflect the higher likelihood that facility staff will attribute missed dialysis sessions to hospitalization, and thus seek medical records, including discharge summaries, from the index hospitalization, among these higher-risk patients. This potentially increased recognition of at-risk patients was associated with higher readmission risk in the absence of a targeted intervention. Together these results suggest that close attention to usual processes of care at the dialysis facility in the post-discharge period may help identify patients at higher risk of readmissions.

Our results do provide some evidence that some interventions at the dialysis facility may be associated with lower readmission risk. Particularly, we found that ≥0.5-kg lower post-dialysis weights in the post- vs. pre-index hospitalization period were associated with substantially reduced risk of pulmonary edema-related readmission risk. Tests of interaction also suggested that the effect was primarily for those readmissions in which the index hospitalization was due to pulmonary edema. Additionally, stratified analyses suggested this effect was strongest among those with a history of CHF. However, it is likely that such changes involved the nephrologist (to review hospital discharges and order changes to the post-dialysis target weight) as well as the dialysis facility staff (to facilitate the change). Thus, these results support the notion that both nephrologists and dialysis facilities are needed to ensure the implementation of processes of care associated with reduced readmission risk [23]. Additionally, while the results were not statistically significant, we did find that index hospitalizations that occurred at facilities with an ultrafiltration rate policy in place were less likely to be followed by a readmission of any type. Such changes to usual processes of care may require facility- or even systems-level policies. In fact, policies to limit the ultrafiltration rate, which will eventually tie performance on these rates to reimbursement, are being introduced into the ESRD Quality Incentive Program for U.S. facilities [24]. While such changes may ultimately improve performance on readmissions, it is likely that these changes will be difficult to operationalize, due to the challenges of increasing dialysis time for even a subset of patients at a facility [25, 26]. Furthermore, for protocols and policies that are aimed at the dialysis facility to result in reduced readmissions, hospitalized dialysis patients would have to return to the dialysis facility immediately after discharge and receive consistent dialysis in the post-discharge period. We found that nearly 1 in 5 dialysis patients discharged from the hospital did not have at least three dialysis sessions within 10 days of discharge. While non-adherence may be a contributing factor to this observation, our data suggest that non-adherence to hemodialysis sessions did not differ substantially between excluded and included groups. Rather, it is likely that many of these patients had already been readmitted within a week of discharge [27].

The primary limitation of our study is the lack of information on events that occurred during the index hospitalization, beyond that available in the administrative data related to dialysis. Thus, we cannot know whether many of the processes of care we are capturing at the dialysis facility are clinically appropriate given hospitalization events. However, information that includes detailed EMR data on both hospitalizations and dialysis sessions in the peri-hospitalization period is difficult to obtain. Most hospitals treat patients from multiple dialysis facilities and patients at most dialysis facilities are admitted to multiple hospitals, with no common EMR systems facilitating communication between dialysis facility and hospital providers, which is necessary for continuity of care. While discharge summaries can and should be sent from the inpatient provider to the outpatient dialysis provider, receipt of a discharge summary by the dialysis facility does not ensure that information on this summary will be timely and noted or entered in any detail into a dialysis facility EMR. In fact, we found that only 63–85% (depending on readmission status) of index hospitalizations we identified in national administrative data were actually documented in the dialysis EMR. This may be partially due to missing or simply untimely [28] discharge summaries. Further, even when discharge summaries are received, they may not contain critical elements for determining appropriateness of post-hospitalization dialysis care, such as updated medication lists (particularly antibiotic use) or dry weight assessments [29].

Other limitations of this study also deserve mention. There is the possibility of selection bias due to the exclusions imposed on our study population, particularly the requirement for primary Medicare coverage and for three dialysis sessions within 10 days of discharge. However, importantly, these exclusions were necessary to ensure we were capturing both index admissions and readmissions, as well as to ensure that we were examining the effects of dialysis facility processes of care only in the cases in which there was adequate time and opportunity for the dialysis facility to assess the patient, contact the nephrologists as necessary, and make changes to the treatment plan. As with any study using EMR and administrative data, misclassification is possible. For example, information on adherence to medications is not available and likely not captured by the ICD-9 code we used to capture noncompliance to dialysis treatments. Also, importantly, some processes of care that potentially affect readmission risk may not be well-documented in an EMR. Residual confounding is also a possibility, as in all observational studies. In our study, confounding by indication could be better controlled if more information on index hospitalization characteristics were available. For example, information on medications provided during the hospitalization and/or prescribed at discharge would help determine the appropriateness of changes after hospitalizations. Finally, our results may not be generalizable to other dialysis populations within or outside the United States. In fact, the readmission risk reported here (18%) is lower than that documented in U.S. national data [1, 27]; however, our proportion of readmissions attributable to pulmonary edema is quite similar to that we reported recently using national data [2]. Our study also has several strengths, including the availability of detailed EMR data from the dialysis facility and the linkage of EMR data to national administrative data to ensure complete capture of hospitalizations. Notably, our study provides a novel, detailed view of the impact of the current usual processes of care in place at dialysis facilities on the risk of hospital readmissions for a large, diverse U.S. in-center hemodialysis population living in urban and rural geographic regions.

## Conclusions

Our results suggest that some interventions performed at the dialysis facility in the post-hospitalization period (e.g., lower post-dialysis weight after hospitalization, limiting ultrafiltration rate) may be associated with reduced readmission risk. Some usual processes of care already in place may help to identify patients at higher risk of readmissions. Overall, these results inform potential targeted interventions in the post-hospitalization period at the dialysis facility to help prevent pulmonary edema-related (representing nearly half of readmissions) or other readmissions, or both. Further studies are needed to better identify and confirm the role of targeted interventions that may help reduce readmissions during the index hospitalization and in the immediate period after hospitalization when the patient returns to the dialysis facility.

## Additional files

Additional file 1 : Table S1 Index admission and patient characteristics of a cohort of prevalent hemodialysis patients admitted at least once while on hemodialysis treatment at Emory or Wake Forest between February 2010 and July 2015, by 30-day readmission of any type and by 30-day pulmonary edema-related readmission. (DOCX 17 kb)

Additional file 2 : Table S2 Associations of dialysis facility processes of care with overall and pulmonary edema-related 30-day readmissions, among hospitalized Emory and Wake Forest hemodialysis patients in 2/2010–7/2015. (DOCX 21 kb)
