# Supplementary material for: Post-hospitalization dialysis facility processes of care and hospital readmissions among hemodialysis patients: a retrospective cohort study
Source: BMC Nephrol. 2018 Jul 31;19:186. doi: 10.1186/s12882-018-0983-5 (PMC6069998; doi:10.1186/s12882-018-0983-5)
Supplement: Supplementary file 1 — : Table S1 Index admission and patient characteristics of a cohort of prevalent hemodialysis patients admitted at least once while on hemodialysis treatment at Emory or Wake Forest between February 2010 and July 2015, by 30-day readmission of any type and by 30-day pulmonary edema-related readmission. (DOCX 17 kb) [file 12882_2018_983_MOESM1_ESM.docx]

***Supplementary Tables/Figures***

**Table S1.** Index admission and patient characteristics of a cohort of prevalent hemodialysis patients admitted at least once while on hemodialysis treatment at Emory or Wake Forest between February 2010 and July 2015, by 30-day readmission of any type and by 30-day pulmonary edema-related readmission

| **Characteristic*** |  | **Any 30-day readmission** | | **Pulmonary edema-related 30-day readmission** | |
| --- | --- | --- | --- | --- | --- |
|  | **Overall** | **No** | **Yes** | **No** | **Yes** |
| *N* | *1056* | *869 (82.3%)* | *187 (17.7%)* | *972 (92.1%)* | *84 (8.0%)* |
| **Index admission** characteristic** |  |  |  |  |  |
| Median length of stay (IQR), days | 4 (2-7) | 4 (2-7) | 4 (2-8) | 4 (2-7) | 4 (2-7) |
| Pulmonary edema-related (%) | 38.8% | 37.7% | 43.9% | 35.6%** | 76.2%** |
| Intensive care utilization (%) | 23.9% | 24.3% | 21.9% | 24.2% | 20.2% |
| **Patient demographics** |  |  |  |  |  |
| Mean (SD) age, years | 60.5 (15.0) | 60.4 (14.9) | 60.3 (15.4) | 60.3 (14.9) | 62.4 (15.4) |
| Female (%) | 47.8% | 47.2% | 50.8% | 47.6% | 50.0% |
| Race/ethnicity (%) |  |  |  |  |  |
| Non-Hispanic white | 33.9% | 33.0% | 38.2% | 32.9% | 45.2% |
| Non-Hispanic Black | 63.5% | 64.2% | 60.2% | 64.3% | 54.8% |
| Hispanic or other | 2.6% | 2.8% | 1.6% | 2.8% | 0.0% |
| **Patient clinical factors** |  |  |  |  |  |
| Median (IQR) dialysis vintage, years | 1.0 (0.3-3.9) | 1.1 (0.3-3.8) | 0.8 (0.2-4.2) | 1.0 (0.3-3.8) | 1.0 (0.3-4.0) |
| History of dialysis non-adherence | 3.4% | 2.6%* | 6.4%* | 3.0% | 7.1% |
| Primary assigned cause of ESRD (%) |  |  |  |  |  |
| Diabetes | 40.9% | 41.2% | 39.0% | 41.1% | 38.1% |
| Hypertension | 27.6% | 27.3% | 28.9% | 27.4% | 29.8% |
| Glomerulonephritis | 11.4% | 11.3% | 11.8% | 11.2% | 13.1% |
| Other | 20.2% | 20.2% | 20.3% | 20.3% | 19.1% |
| Comorbid conditions (%): |  |  |  |  |  |
| Diabetes | 59.8% | 60.3% | 57.8% | 60.3% | 54.8% |
| Ischemic heart disease | 27.3% | 27.1% | 28.3% | 26.6% | 34.5% |
| Hypertension | 98.9% | 98.8% | 99.5% | 98.9% | 98.8% |
| Congestive heart failure | 42.4% | 40.8% | 48.7% | 39.2%** | 73.8%** |

BMI, body mass index; COPD, chronic obstructive pulmonary disease; ESRD, end-stage renal disease, IQR, interquartile range. *N*=1056 overall, except for race/ethnicity (*N*=1050), assigned cause of ESRD (*N*=1055).

**P*<0.05; ***P*<0.001, by Fisher’s exact.
