# Supplementary material for: Post-hospitalization dialysis facility processes of care and hospital readmissions among hemodialysis patients: a retrospective cohort study
Source: BMC Nephrol. 2018 Jul 31;19:186. doi: 10.1186/s12882-018-0983-5 (PMC6069998; doi:10.1186/s12882-018-0983-5)
Supplement: Supplementary file 2 — : Table S2 Associations of dialysis facility processes of care with overall and pulmonary edema-related 30-day readmissions, among hospitalized Emory and Wake Forest hemodialysis patients in 2/2010–7/2015. (DOCX 21 kb) [file 12882_2018_983_MOESM2_ESM.docx]

***Supplementary Tables/Figures***

**Table S2.** Associations of dialysis facility processes of care with overall and pulmonary edema-related 30-day readmissions, among hospitalized Emory and Wake Forest hemodialysis patients in 2/2010-7/2015

| **Dialysis facility process of care*** | **Any readmission** | | | **Pulmonary edema-related readmission** | | |
| --- | --- | --- | --- | --- | --- | --- |
|  | **No** | **Yes** | ***P*** | **No** | **Yes** | ***P*** |
| Index admission documented |  |  |  |  |  |  |
| % documented | 62.5% | 82.4% | *<0.001* | 64.4% | 84.5% | *<0.001* |
| Unadjusted OR (95% CI) | 1.00 (ref.) | 2.80 (1.88-4.18) | *<0.001* | 1.00 (ref.) | 3.02 (1.65-5.53) | *<0.001* |
| Adjusted** OR (95% CI) | 1.00 (ref.) | 1.71 (1.12-2.60) | *0.01* | 1.00 (ref.) | 1.95 (1.03-3.70) | *0.04* |
| % patients with CHF documented in problem list at index discharge (among n=389 patients with CHF history) |  |  |  |  |  |  |
| % yes | 39.9% | 44.0% | *0.5* | 37.9% | 56.5% | *0.007* |
| Unadjusted OR (95% CI) | 1.00 (ref.) | 1.18 (0.73-1.90) | *0.5* | 1.00 (ref.) | 2.12 (1.23-3.68) | *0.007* |
| Adjusted** OR (95% CI) | 1.00 (ref.) | 1.20 (0.74-1.93) | *0.5* | 1.00 (ref.) | 1.97 (1.13-3.45) | *0.02* |
| % any labs drawn within 3 sessions after index discharge |  |  |  |  |  |  |
| % yes | 65.7% | 82.4% | *<0.001* | 67.4% | 83.3% | *0.003* |
| Unadjusted OR (95% CI) | 1.00 (ref.) | 2.44 (1.63-3.64) | *<0.001* | 1.00 (ref.) | 2.42 (1.34-4.36) | *0.003* |
| Adjusted** OR (95% CI) | 1.00 (ref.) | 1.35 (0.89-2.05) | *0.2* | 1.00 (ref.) | 1.35 (0.73-2.49) | *0.3* |
| % albumin checked within 3 sessions after index discharge |  |  |  |  |  |  |
| % yes | 33.0% | 43.9% | *0.005* | 34.8% | 36.9% | *0.7* |
| Unadjusted OR (95% CI) | 1.00 (ref.) | 1.58 (1.15-2.18) | *0.005* | 1.00 (ref.) | 1.10 (0.69-1.74) | *0.7* |
| Adjusted** OR (95% CI) | 1.00 (ref.) | 1.23 (0.89-1.71) | *0.2* | 1.00 (ref.) | 0.84 (0.52-1.35) | *0.5* |
| % hemoglobin/hematocrit checked within 3 sessions after index discharge |  |  |  |  |  |  |
| % yes | 59.4% | 78.1% | *<0.001* | 61.2% | 79.8% | *0.001* |
| Unadjusted OR (95% CI) | 1.00 (ref.) | 2.44 (1.68-3.53) | *<0.001* | 1.00 (ref.) | 2.50 (1.44-4.32) | *0.001* |
| Adjusted** OR (95% CI) | 1.00 (ref.) | 1.53 (1.04-2.24) | *0.03* | 1.00 (ref.) | 1.60 (0.91-2.83) | *0.1* |
| % decrease in post-dialysis weight in the first 3 sessions of at least 0.5 kg |  |  |  |  |  |  |
| % yes | 56.6% | 52.9% | *0.4* | 56.5% | 50.0% | *0.3* |
| Unadjusted OR (95% CI) | 1.00 (ref.) | 0.86 (0.63-1.18) | *0.4* | 1.00 (ref.) | 0.77 (0.49-1.20) | *0.3* |
| Adjusted** OR (95% CI) | 1.00 (ref.) | 0.84 (0.61-1.17) | *0.3* | 1.00 (ref.) | 0.59 (0.37-0.95) | *0.03* |
| % higher ESA dose ordered in first 3 sessions (among n=282 with ESA administered) |  |  |  |  |  |  |
| % yes | 16.7% | 11.7% | *0.3* | 15.7% | 15.0% | *>0.9* |
| Unadjusted OR (95% CI) | 1.00 (ref.) | 0.66 (0.28-1.57) | *0.3* | 1.00 (ref.) | 0.95 (0.27-3.39) | *>0.9* |
| Adjusted** OR (95% CI) | 1.00 (ref.) | 0.67 (0.28-1.60) | *0.4* | 1.00 (ref.) | 0.95 (0.26-3.50) | *>0.9* |
| % any home medication discontinued in first 3 sessions |  |  |  |  |  |  |
| % yes | 16.1% | 25.1% | *0.004* | 16.6% | 31.0% | *0.002* |
| Unadjusted OR (95% CI) | 1.00 (ref.) | 1.75 (1.20-2.55) | *0.004* | 1.00 (ref.) | 2.26 (1.38-3.70) | *0.001* |
| Adjusted** OR (95% CI) | 1.00 (ref.) | 1.46 (1.00-2.15) | *0.05* | 1.00 (ref.) | 1.79 (1.07-3.00) | *0.03* |
| % any home medication changed in first 3 sessions |  |  |  |  |  |  |
| % yes | 27.6% | 29.9% | *0.5* | 26.8% | 41.2% | *0.003* |
| Unadjusted OR (95% CI) | 1.00 (ref.) | 1.12 (0.81-1.54) | *0.5* | 1.00 (ref.) | 1.92 (1.25-2.94) | *0.003* |
| Adjusted** OR (95% CI) | 1.00 (ref.) | 1.11 (0.79-1.55) | *0.6* | 1.00 (ref.) | 1.74 (1.09-2.76) | *0.002* |
| % index discharge occurring at facility with current ultrafiltration rate policy*** |  |  |  |  |  |  |
| % yes | 30.0% | 19.3% | *0.003* | 28.9% | 19.1% | *0.05* |
| Unadjusted OR (95% CI) | 1.00 (ref.) | 0.56 (0.38-0.82) | *0.003* | 1.00 (ref.) | 0.58 (0.33-1.01) | *0.06* |
| Adjusted** OR (95% CI) | 1.00 (ref.) | 0.78 (0.52-1.16) | *0.2* | 1.00 (ref.) | 0.77 (0.43-1.38) | *0.4* |

CHF, congestive heart failure; ESA, erythropoietin-stimulating agent.

*Assessed in the first three sessions after index discharge.

**Adjusted models include patient history of congestive heart failure, index admission related to pulmonary edema, and history of non-adherence.

****Ultrafiltration rate policy required providers to lengthen prescribed treatment time in increments of 15 minutes (up to 1 hour) for any session in which the patient’s anticipated ultrafiltration rate (given intradialytic weight gain) was >13 ml/kg/hour.
